# Clinical Trials and Emerging Therapeutic Paradigms in Upper-Tract Urothelial Carcinoma

**DOI:** 10.3390/cancers18081223

**Published:** 2026-04-13

**Authors:** Julian Chavarriaga, Jay D. Raman

**Affiliations:** Department of Urology, Penn State Health, Hershey, PA 17033, USA

**Keywords:** upper-tract urothelial carcinoma, chemotherapy, immunotherapy, urothelial cancer, survival, kidney preservation

## Abstract

Upper-tract urothelial carcinoma (UTUC) represents a biologically distinct and clinically challenging subset of urothelial malignancies, accounting for only 5–10% of urothelial cancers but carrying a disproportionately high risk of advanced disease and recurrence. Historically, management strategies for UTUC have been extrapolated from bladder cancer data, with limited prospective evidence specific to the upper urinary tract. However, recent years have witnessed an expanding number of UTUC-focused clinical trials that are reshaping treatment paradigms across localized, locally advanced, and metastatic disease states. This review examines the evolving landscape of clinical trials in UTUC, highlighting pivotal and ongoing studies that will inform contemporary management. We summarize evidence supporting perioperative systemic therapy, including neoadjuvant and adjuvant chemotherapy, and discuss the expanding role of immune checkpoint inhibitors in both perioperative and metastatic settings. Additionally, we review trials evaluating kidney-sparing approaches, intraluminal therapies, and novel drug-delivery platforms aimed at preserving renal function while maintaining oncologic control. Emerging trial designs incorporating molecular profiling, fibroblast growth factor receptor (FGFR)-targeted therapies, and biomarker-driven patient selection are also explored. Despite meaningful progress, significant gaps remain, including the underrepresentation of UTUC patients in large urothelial cancer trials, heterogeneity in risk stratification, and challenges in trial accrual for this rare disease. We conclude by outlining future directions for UTUC-specific clinical research, emphasizing the need for collaborative, multicenter trials, innovative endpoints, and integrated translational studies to further refine personalized treatment strategies. As the clinical trial ecosystem for UTUC continues to mature, these efforts hold promises for improving outcomes while balancing oncologic efficacy with renal preservation.

## Simple Summary

Upper-tract urothelial carcinoma (UTUC) is a rare cancer that arises in the lining of the kidney and ureter. Although it represents only 5–10% of urothelial cancers, it often presents at an advanced stage and can be difficult to treat. Historically, treatment decisions have been based on research from bladder cancer rather than UTUC-specific studies. This review summarizes recent and ongoing clinical trials that are improving how UTUC is managed. These include studies of chemotherapy, immunotherapy, kidney-sparing treatments, and new targeted therapies.

## 1. Introduction

Upper-tract urothelial carcinoma (UTUC) is an uncommon malignancy arising from the urothelial lining of the renal pelvis and ureter, accounting for approximately 5 to 10% of all urothelial carcinomas. In the United States, an estimated 7000 cases are diagnosed annually, based on American Cancer Society projections and Surveillance, epidemiology, and end results (SEER) data, corresponding to an incidence of roughly 0.9 to 1.0 cases per 100,000 population. Although histologically similar to urothelial carcinoma of the bladder, UTUC demonstrates distinct epidemiologic, molecular, and clinical features. Notably, approximately 60% of UTUCs are invasive at diagnosis, compared with 15–25% of bladder tumors, and outcomes are generally poorer even when comparing equivalent stages [1,2,3]. 

Management remains challenging. Patients with UTUC are typically elderly, comorbid, and harbor baseline renal impairment. As such, many are limited in their ability to received cisplatin-based chemotherapy. Furthermore, although radical nephroureterectomy is the standard surgical treatment, it often is associated with perioperative complications and results in further deterioration of kidney function. Collectively, these features distinguish UTUC from bladder cancer and underscore the need for disease specific therapeutic strategies rather than direct extrapolation from bladder cancer paradigms [3]. 

The optimal management of UTUC relies on multidisciplinary expertise spanning urologic surgery, medical oncology, pathology, radiology, and genetics, ideally within experienced referral centers. In recent years, advances in molecular profiling, biomarker development, perioperative systemic therapy, nephron-sparing approaches, and novel drug platforms have begun to reshape the treatment landscape. This article reviews contemporary clinical trials and emerging therapeutic paradigms in UTUC, with a focus on biomarker-driven risk stratification, neoadjuvant and adjuvant treatment strategies, evolving approaches to kidney preservation, advances in metastatic disease management, and promising future directions that may further individualize care.

## 2. Materials and Methods

We searched PubMed for available literature on upper-tract urothelial carcinoma (UTUC), focusing on emerging biomarkers, neoadjuvant and adjuvant systemic therapies, nephron-sparing strategies, and advances in metastatic disease management. Search terms included “upper tract urothelial carcinoma,” “UTUC,” “circulating tumor DNA,” “neoadjuvant therapy,” “adjuvant therapy,” “chemotherapy,” “immunotherapy,” and “antibody–drug conjugates”. We only included articles published within 20 years of this publication. Articles published in English and Spanish were included. Non-human studies, editorials, letters, and case reports were excluded. In addition, we reviewed recently published clinical trials, conference proceedings, and contemporary clinical practice guidelines relevant to UTUC. Reference lists from key guidelines and pivotal trials were screened to identify additional relevant studies. This narrative review was conducted to ensure inclusion of both foundational and emerging evidence informing evolving therapeutic strategies in UTUC. Given the narrative nature of this review, we must acknowledge there may be some inherent selection bias.

## 3. Results

### 3.1. Emerging Biomarkers for UTUC

Accurate risk stratification before radical nephroureterectomy remains a major challenge in UTUC, because clinical stage is often underestimated and key pathological features are only confirmed after surgery. As a result, preoperative risk tools have been developed to aid treatment selection and clinical trial enrollment. The nomogram reported by Petros and colleagues incorporates preoperative patient characteristics together with imaging, endoscopic, and laboratory variables, and has shown good accuracy for predicting locally advanced, non-organ-confined UTUC [4]. Such models can help identify patients most likely to benefit from perioperative systemic therapy, but they remain limited by the inherent constraints of preoperative sampling and imaging.

There is therefore a need for biomarkers that are diagnostic, minimizing procedural burden; predictive, identifying patients most likely to benefit from therapies such as neoadjuvant chemotherapy; and prognostic, improving risk stratification.

Cell-free DNA derived from plasma or urine is an attractive candidate. Its accessibility and short half life allow dynamic assessment of tumor biology in real time [4]. In bladder urothelial carcinoma, tumor-derived DNA in circulation has shown good concordance with tissue alterations, and both the presence and level of alterations correlate with disease burden and prognosis, supporting investigation of this approach in UTUC [5]. Circulating tumor DNA (ctDNA) has emerged as a complementary strategy to improve clinical staging by capturing tumor biology that may not be apparent on conventional evaluation. In bladder cancer, absence of preoperative circulating tumor DNA has been associated with excellent postoperative outcomes, suggesting that ctDNA may help better identify patients who are most likely to benefit from neoadjuvant therapy in urothelial malignancies [3,6]. In UTUC, a prospective study led by Huelster evaluated chemotherapy-naïve high-risk patients undergoing upfront surgery detected ctDNA variants in 70% of cases. ctDNA positivity was more frequently observed in patients with muscle-invasive or non-organ-confined disease. Detectable preoperative ctDNA was associated with poorer prognosis and worse oncologic outcomes. Although concordance between plasma and tissue alterations was not complete, these findings support further investigation of ctDNA as a prognostic and staging tool in UTUC. Such approaches may ultimately help guide treatment selection, including the choice between upfront surgery and neoadjuvant systemic therapy for patients with upper-tract urothelial carcinoma [7]. 

Urinary biomarkers have been developed to overcome the limitations of cytology and ureteroscopic biopsy in UTUC, particularly for preoperative risk assessment [8]. UroVysion (Des Plaines, IL, USA) fluorescence in situ hybridization (FISH) detects chromosomal abnormalities involving chromosomes 3, 7, and 17, as well as loss of 9p21. Reported sensitivity ranges from 35% to 100%, while specificity is generally between 80% and 100%. Test performance is highest in high-grade disease and when upper-tract samples are used [9,10]. However, its ability to discriminate tumor grade remains limited. ADXBLADDER (Sunderland, England), which detects the MCM5 protein, has shown moderate sensitivity in urothelial carcinoma with relatively high specificity and negative predictive value, though dedicated UTUC data remain limited [9,10]. 

Epigenetic and transcript-based assays have demonstrated more promising results. Bladder EpiCheck (San Diego, CA, USA), a 15-gene methylation assay, has shown sensitivity approaching 97% for high-grade UTUC in surgical cohorts, with pooled analyses reporting sensitivity around 85% and specificity exceeding 90%. Xpert BC Detection (Sunnyvale, CA, USA), an mRNA-based assay, has demonstrated very high sensitivity in small UTUC series but poor specificity, limiting its current clinical applicability. Collectively, these assays highlight the growing role of molecular diagnostics in UTUC, although most require further validation before routine integration into clinical decision making [8]. 

### 3.2. Neoadjuvant Therapy for UTUC

Radical nephroureterectomy remains the standard treatment for UTUC, yet outcomes for patients with adverse pathologic features are poor. Five-year cancer specific survival exceeds 90% for non-muscle-invasive tumors but declines to approximately 40% for patients with pT3 disease. For example, in a cohort of 414 UTUC patients undergoing RNU, 43% had locally advanced tumors, with three- and five-year cancer specific survival of 47% and 34%, respectively [11]. Despite this high risk, only 31% received adjuvant chemotherapy. Postoperative decline in renal function and surgical morbidity likely limited the use of adjuvant chemotherapy. Mean estimated glomerular filtration rate decreased from 59 to 51 mL/min/1.73 m^2^, with approximately one quarter of patients falling below cisplatin eligibility thresholds, and postoperative complications occurring in 26% of patients. Although adjuvant chemotherapy was associated with a 15% improvement in cancer specific survival, many patients were unable to receive optimal therapy after surgery. These data support consideration of neoadjuvant chemotherapy, when renal function is preserved and treatment delivery is more feasible, while underscoring the importance of accurate preoperative risk stratification in UTUC [3,11]. Notably, most data in the neoadjuvant setting are derived from retrospective cohorts or phase II prospective studies. To date, no phase III randomized controlled trial has demonstrated superiority of this approach.

Prospective phase 2 studies have demonstrated encouraging activity and acceptable safety of neoadjuvant chemotherapy in high-risk UTUC. In ECOG-ACRIN 8141, accelerated MVAC administered before radical nephroureterectomy led to pathologic downstaging to <ypT2 in 62% of patients, with a complete response in 13.8%. Renal function declined minimally after chemotherapy but substantially after surgery, reinforcing the rationale for delivering cisplatin-based therapy preoperatively [12]. Similar findings were observed in a multicenter phase 2 trial of neoadjuvant gemcitabine plus split-dose cisplatin in 57 patients, in which 63% achieved <ypT2 and 19% had ypT0N0. Importantly, patients who achieved a pathologic response had significantly longer progression-free and overall survival compared with non-responders [13]. Long-term follow-up from Jayalath and colleagues further supports this treatment approach [13,14]. In the original cohort of 57 patients treated with gemcitabine and split-dose cisplatin, the pathologic response rate remained 63%. With a median follow-up of 5.4 years, the estimated 7-year disease-free, cancer-specific, and overall survival rates were 60%, 77%, and 72%, respectively. Outcomes were markedly better among responders to neoadjuvant therapy, with 7-year disease-free survival of 78% versus 31% in non-responders, cancer-specific survival of 90% versus 56%, and overall survival of 87% versus 48%. These findings were confirmed in an expanded institutional cohort of 126 patients and represent the most mature prospective data supporting neoadjuvant cisplatin-based chemotherapy in high-risk non-metastatic UTUC.

Despite the promising data highlighted above, it is important to recognize that early phase studies have yielded mixed results. In the phase 2 PURE-02 trial, neoadjuvant pembrolizumab monotherapy was administered to ten patients with high-grade UTUC; only one patient achieved <ypT2N0 at surgery, and 20% required addition of platinum-based chemotherapy because of progression during treatment [13]. Similarly, the phase 2 iNDUCT-GETUG V08 trial assessed neoadjuvant durvalumab combined with platinum-based chemotherapy. In the cisplatin arm of 29 patients, the ypT0 rate was 13% and 52% achieved <ypT2N0, although the prespecified target complete response rate of 25% was not met [14]. A summary of the studies in the neoadjuvant space is presented in Table 1.

There is increasing interest in refining neoadjuvant strategies for UTUC through incorporation of immunotherapy and novel systemic agents. An ongoing randomized phase 3 trial is evaluating whether the addition of durvalumab to accelerated MVAC before radical nephroureterectomy improves event free survival compared with chemotherapy alone (NCT04628767). Beyond these approaches, enfortumab vedotin plus pembrolizumab, now a frontline standard in advanced urothelial carcinoma [15,16] and in cisplatin-ineligible MIBC [17], as a perioperative treatment strategy with limited nephrotoxicity, is being evaluated in the perioperative setting for UTUC (NCT05775471). Together, these studies reflect an evolving effort to enhance pathological response while preserving renal function and expanding eligibility for systemic therapy.

**Table 1:** Neoadjuvant therapy studies for upper-tract urothelial carcinoma (UTUC).

Trial	Phase	Regimen	Patients	Cycles Planned	ypT0N0	<ypT2N0
**ECOG-ACRIN 8141 (NCT02412670)** [12]	2	Accelerated MVAC (CrCl > 50 mL/min)	30	4	14% *	62% **
**NCT01261728 (MSKCC)** [18]	2	Gemcitabine + split-dose cisplatin (CrCl > 55 mL/min)	57	4	19%	63%
**PURE-02 (NCT02736266)** [13]	2	Pembrolizumab	10	3	14%	28%
**iNDUCT-GETUG V08 (NCT04617756)** [14]	2	Durvalumab + gemcitabine + cisplatin (CrCl > 60 mL/min): Cohort 1	30	4	13%	50% **
**NCT01261728 Pooled Expanded cohort (2026)** [19]	2 (phase 2 + expanded institutional cohort)	Gemcitabine + split-dose cisplatin (CrCl > 55 mL/min)	126	Up to 4	16%	67%

* 3 achieved ypT0N0 (10.3%), and a fourth achieved ypT0Nx (pCR = 13.8%). ** Final pathologic stage ≤ pT1N0.

#### Perioperative Therapy for UTUC

In muscle-invasive urothelial carcinoma of the bladder, perioperative combination strategies have clearly reshaped the treatment landscape. In the NIAGARA trial, cisplatin-eligible patients with cT2–T4aN0/1M0 disease were randomized to receive neoadjuvant durvalumab combined with cisplatin and gemcitabine followed by radical cystectomy and adjuvant durvalumab, or chemotherapy and surgery alone. Durvalumab reduced the risk of distant metastases or death by 33% (HR 0.67) and reduced the risk of death by 31% (HR 0.69), establishing perioperative chemoimmunotherapy as a new standard [20]. In KEYNOTE-905, cisplatin-ineligible patients were randomized to one of three arms: perioperative pembrolizumab alone, perioperative enfortumab vedotin plus pembrolizumab, or radical cystectomy with observation. In the enfortumab vedotin plus pembrolizumab arm, patients received three cycles of neoadjuvant therapy followed by surgery and then continued adjuvant pembrolizumab with additional enfortumab vedotin. Pathologic complete response was a key secondary endpoint and was markedly higher with the combination, 57.1% versus 8.6% with surgery alone, and this translated into a 50% reduction in the risk of death (HR 0.50) [21]. 

Similarly, KEYNOTE B15 (EV 304) demonstrated improved outcomes with neoadjuvant enfortumab vedotin plus pembrolizumab compared with standard cisplatin-based chemotherapy in eligible patients, further reinforcing the shift toward antibody–drug conjugate and immunotherapy combinations in the perioperative setting [22]. Together, these studies have fundamentally changed how we approach muscle-invasive bladder cancer. However, no patients with UTUC were included in these trials. Although extrapolation to selected high-risk UTUC cases may be biologically reasonable, there is currently no prospective data supporting routine use of these perioperative combinations in UTUC, and dedicated studies are needed before incorporation into standard practice.

### 3.3. Adjuvant Therapy for UTUC

Adjuvant systemic therapy for UTUC has been most clearly defined by the POUT trial, a randomized phase 3 study evaluating platinum-based chemotherapy after radical nephroureterectomy in patients with pT2–T4 or node positive disease. POUT demonstrated a significant improvement in disease-free survival with adjuvant chemotherapy compared with surveillance, establishing postoperative platinum-based therapy as a standard for high-risk UTUC [23]. Benefit was observed across subgroups, including patients treated with cisplatin or carboplatin, although cisplatin remains preferred when renal function allows. These results provided the first level I evidence supporting multimodal management in UTUC and directly informed contemporary guideline recommendations [3]. 

Subsequent phase 3 trials of adjuvant immunotherapy in high-risk urothelial carcinoma have included patients with upper-tract primary tumors. CheckMate 274 demonstrated improved disease-free survival with adjuvant nivolumab compared with placebo after radical surgery, including a UTUC subset [24]. In contrast, IMvigor 010 did not show a disease-free survival benefit with adjuvant atezolizumab in the overall population, although exploratory analyses suggested potential benefit in patients with detectable ctDNA [5,25]. The more recent AMBASSADOR trial reported improved disease-free survival with adjuvant pembrolizumab versus observation, again including patients with UTUC [26]. Building on the biomarker-driven signal observed in IMvigor 010, IMvigor 011 prospectively evaluated adjuvant atezolizumab in patients with detectable ctDNA after radical surgery and demonstrated improved disease-free survival in this molecularly selected population [5,25]. Although none of these immunotherapy trials were exclusive to UTUC, their inclusion of upper-tract cases supports risk adapted incorporation of adjuvant checkpoint blockade in selected high-risk UTUC patients (see Table 2).

### 3.4. NSSs and Adjuvant Therapy

Nephron-sparing strategies (NSSs) play an important role in the management of upper-tract urothelial carcinoma, particularly in patients with low-risk favorable disease. In these patients, endoscopic tumor ablation should be considered as first-line therapy when technically feasible [3]. Observational studies have shown cancer-specific survival comparable to radical nephroureterectomy, while better preserving renal function.

Tumor ablation may also be considered in carefully selected patients with low-risk unfavorable UTUC and in certain patients with high-risk favorable disease, particularly when tumors are low volume or when patients are not suitable candidates for radical nephroureterectomy. Although the available evidence is largely retrospective and subject to selection bias, nephron-sparing approaches provide a strategy to balance oncologic control with renal preservation, which may be especially important for patients whose future eligibility for systemic therapy could be compromised by nephron loss.

Chemoablation has become increasingly incorporate as an NSS for UTUC. The OLYMPUS trial was an open-label, single-arm, phase 3 study evaluating intracavitary chemoablation with UGN 101, a mitomycin containing reverse thermal gel, in patients with biopsy proven low-grade UTUC measuring 5 to 15 mm. Among 71 treated patients, 59% achieved a complete response following six weekly instillations delivered retrograde to the renal pelvis and calyces. With longer-term follow-up, 56% of complete responders remained disease free at 12 months, with a Kaplan–Meier estimated durability of 82%, supporting clinically meaningful response persistence with or without maintenance therapy. However, treatment related toxicity was notable, particularly ureteric stenosis in 44% of patients, along with urinary tract infection, hematuria, and flank pain, and 27% experienced serious adverse events. These findings establish UGN 101 as an effective kidney-sparing option for selected patients with low-grade UTUC, while highlighting the need for careful surveillance and management of ureteral complications [28,29]. 

The ongoing uTRACT registry is evaluating real world outcomes following FDA approval of UGN 101. This multicenter registry aims to enroll approximately 400 adults with UTUC treated with at least one dose, capturing both retrospective and prospective data across roughly 20 sites in the United States. Key outcomes include initial complete response, duration of response, recurrence free survival, time to progression, and adverse events. As of May 2025, 274 patients have been enrolled across 22 centers, and these data are expected to further define the durability, safety profile, and generalizability of chemoablation in routine practice [30]. 

Building on the limitations observed with mitomycin hydrogel, particularly the substantial rate of ureteral stricture and limited global availability, alternative chemoablative platforms are being actively explored. An ongoing investigator initiated multicenter trial is evaluating ST 02, a mucoadhesive gemcitabine polymer suspension designed for chemoablation in low-grade UTUC (NCT06124976). ST 02 delivers gemcitabine within a water-free polymer that transitions to a gel upon contact with urine, enhancing urothelial adherence and prolonging local drug exposure. In parallel, a phase 3 single-arm multicenter study assesses UGN 104, a novel formulation derived from UGN 101, in patients with low-grade UTUC. Additionally, the phase 1/2 LUNAR trial (NCT06668493) evaluates nadofaragene firadenovec, a replication deficient adenoviral vector delivering interferon alpha 2b, administered into the renal pelvis via balloon catheter every three months in patients with low-grade UTUC.

An additional emerging strategy for high-risk UTUC is being explored in the phase II WUTSUP 04 trial, which evaluates perioperative enfortumab vedotin combined with toripalimab followed by an endoscopic surgery. This prospective study enrolls patients with non-metastatic high-risk UTUC who have imperative or strong indications for nephron preservation, aiming to downstage tumors with systemic therapy before local ablation and consolidation treatment. The primary endpoint is conversion free survival, defined by avoidance of radical nephroureterectomy.

### 3.5. Treatment Paradigms in Systemic Therapy for Metastatic Urothelial Carcinoma (mUC)

The modern systemic treatment paradigm for mUC integrates immune checkpoint inhibition and targeted therapy, with key phase 3 trials including patients with UTUC. In the CheckMate 901 trial, the anti-PD-1 antibody nivolumab was combined with first-line gemcitabine and cisplatin, with overall survival as the primary endpoint. The combination resulted in a 28% reduction in the risk of progression or death [31]. Moreover, in JAVELIN Bladder 100, the anti-PD-L1 antibody avelumab was evaluated as switch maintenance therapy after platinum-based chemotherapy in patients without progression. The primary endpoint of overall survival was significantly improved, with median overall survival of 23.8 months in the avelumab group versus 18 months, supporting early maintenance immunotherapy rather than observation after chemotherapy [32]. 

For patients with susceptible fibroblast growth factor receptor (FGFR)2 or FGFR3 alterations, the THOR trial evaluated the oral FGFR inhibitor erdafitinib in the post platinum setting, with overall survival as the primary endpoint. Erdafitinib significantly improved median overall survival compared with chemotherapy, 12.1 months versus 7.8 months, corresponding to a hazard ratio for death of 0.64. Progression-free survival was also prolonged with targeted therapy. Given the higher prevalence of FGFR3 alterations in UTUC compared with bladder carcinoma, these data underscore the importance of early genomic testing and support incorporation of biomarker-directed therapy into the treatment sequence [33]. Collectively, these trials outline a therapeutic framework for metastatic UTUC that prioritizes cisplatin-based chemoimmunotherapy when feasible, maintenance PD-L1 blockade for patients with response or stable disease, and FGFR inhibition in appropriately selected patients with actionable alterations.

### 3.6. Antibody–Drug Conjugates in Urothelial Carcinoma: Bridging Innovation and Clinical Applicability

**Figure 1:**
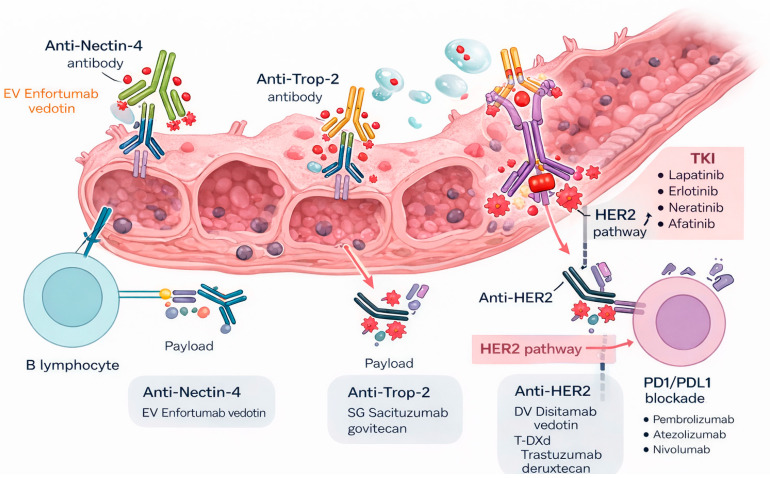
Antibody–drug conjugates (ADCs) and immune checkpoint inhibition in upper-tract urothelial carcinoma (UTUC). EV: Enfortumab vedotin; SG: Scituzumab govitecan; PD1/PDL1: Programmed death 1/programmed death ligand 1; TKI: Tyrosine kinase inhibitors; DV: Disitimab vedotin; T-DXd: trastuzumab deruxtecan

Antibody–drug conjugates are targeted cytotoxic therapies built from three linked components: a monoclonal antibody that binds a tumor associated cell surface antigen, a linker designed to remain stable in circulation but release drug in the tumor microenvironment or after internalization, and a potent payload that delivers the cytotoxic effect. In urothelial carcinoma, the most clinically relevant ADC targets include Nectin 4, Trop 2, and HER2, reflecting antigens with relatively high tumor expression and established therapeutic activity in advanced disease [15,16,21,22,34,35] (see Figure 1).

The strongest ADC data in urothelial carcinoma come from enfortumab vedotin, a Nectin 4-directed ADC [15,16,21,22]. In EV 301, enfortumab vedotin improved overall survival versus chemotherapy in previously treated advanced urothelial carcinoma, with a 30% reduction in the risk of death (HR 0.70, 95% CI 0.58 to 0.85) [16]. In the first line setting, EV 302 confirmed the benefit of combining enfortumab vedotin with pembrolizumab, with improvements in progression-free survival (HR 0.48, 95% CI 0.41 to 0.57) and overall survival (HR 0.51, 95% CI 0.43 to 0.61), and benefit reported across key subgroups including cisplatin eligibility and liver metastases [15]. 

Other ADC targets highlight both promise and limitations. Sacituzumab govitecan, a Trop 2-directed ADC, demonstrated activity in the phase 2 TROPHY U 01 study with an objective response rate of 28% and median progression-free and overall survival of 5.4 and 10.9 months, respectively [35]. However, the phase 3 TROPiCS 04 trial did not meet its primary endpoint, with an median overall survival of 10.3 months versus 9.0 months for treatment of physician’s choice (HR 0.86, 95% CI 0.73 to 1.02) [36]. HER2-directed ADC strategies are also expanding. Trastuzumab deruxtecan has shown activity across HER2-mutated solid tumors in the DESTINY PanTumor01 basket study [37]. Phase 2 studies have demonstrated promising activity of disitamab vedotin in HER2-expressing urothelial carcinoma. This agent is now being investigated in UTUC-specific kidney-sparing strategies, such as the DISTINCT I trial, which combines disitamab vedotin with tislelizumab followed by nephron-sparing surgery (ureteroscopic tumor ablation or segmental ureterectomy) [34]. 

Collectively, antibody–drug conjugates have redefined the therapeutic landscape of advanced urothelial carcinoma and are rapidly moving into earlier disease settings. Their ability to deliver potent cytotoxic therapy selectively to tumor cells, while leveraging biologically relevant targets such as Nectin 4, Trop 2, and HER2, has translated into meaningful survival gains. For UTUC, where distinct molecular features such as FGFR3 alterations and HER2 expression are relatively common, ADC-based strategies may be particularly relevant. Ongoing trials exploring perioperative and kidney-sparing applications will determine whether these agents can further expand beyond metastatic disease and meaningfully alter the natural history of UTUC.

### 3.7. Future Directions in UTUC

#### 3.7.1. Photodynamic Therapy

The ENLIGHTED phase III trial is a global, single-arm study evaluating padeliporfin vascular-targeted photodynamic therapy for patients with low-grade, non-invasive UTUC. This approach involves intravenous administration of the photosensitizer padeliporfin followed by targeted activation with near-infrared laser light delivered endoscopically. Light activation generates reactive oxygen species within the tumor microvasculature, leading to endothelial injury, vascular occlusion, and ischemic tumor necrosis. Because the photosensitizer remains largely confined to the vascular compartment and light delivery is spatially targeted, tumor destruction occurs with limited injury to surrounding urothelial tissue. Among 79 evaluable patients in the interim analysis, overall response was 86.5%, including a 73% complete response rate after induction therapy, with low rates of recurrence and progression. Treatment was generally well tolerated, with most adverse events being grade 1 to 2 and transient; grade 3 events were uncommon and manageable. These findings suggest that photodynamic therapy may offer an effective, kidney-sparing alternative for selected patients with low-grade UTUC, particularly those seeking to avoid nephroureterectomy, although longer follow-up is needed to confirm durability and define its role within contemporary treatment algorithms [38]. 

#### 3.7.2. Drug-Delivery Devices

The TAR 200 system is a small, coin sized, pretzel shaped intravesical device designed to provide sustained local delivery of gemcitabine within the bladder over several weeks, minimizing systemic exposure while maintaining continuous intraluminal drug concentrations. In the SunRISe 1 trial in BCG unresponsive high-risk non-muscle-invasive bladder cancer, TAR 200 monotherapy achieved a complete response rate of 82.4%, with a median duration of response of 25.8 months. The broader SunRISe program includes SunRISe 4 evaluating TAR 200 plus cetrelimab in cisplatin-ineligible muscle-invasive disease, SunRISe 5 in high-risk BCG exposed non-muscle-invasive disease, and related MoonRISe studies assessing TAR 210, an erdafitinib eluting device for FGFR altered tumors. Although developed for bladder cancer, this platform highlights the potential of sustained intraluminal drug-delivery strategies that could be adapted to UTUC in the future.

#### 3.7.3. Opportunity for Radioligand Therapy (RLT) in Urothelial Cancer

Fibroblast activation protein-targeted imaging represents an emerging theranostic platform in urothelial carcinoma. Radiotracers such as ^68^Ga DOTA FAPI and ^68^Ga FAP 2286 enable PET-based visualization of tumor associated fibroblast activation protein expression, while FAP 2286, a cyclic peptide with high affinity for fibroblast activation protein, can be conjugated to Lutetium-177 to deliver targeted radioligand therapy [39]. Early clinical experience with ^68^Ga FAP 2286 PET imaging in urothelial carcinoma has demonstrated tumor uptake and feasibility, and parallel translational work using ^68^Ga labeled tracers targeting Nectin 4 further supports the concept of antigen-directed imaging in this disease [40]. Given the distinct biology of UTUC and the need for improved staging and systemic treatment strategies, fibroblast activation protein and other target-based theranostic approaches represent a promising area for future research in UTUC, with potential applications in both advanced and high-risk localized disease [39].

While these emerging strategies highlight the rapid evolution of the UTUC therapeutic landscape, future research should move beyond conceptual frameworks toward more structured and actionable approaches. Specifically, there is a critical need for prospective, UTUC-specific clinical trials with standardized eligibility criteria, endpoints, and reporting metrics to allow for meaningful cross-study comparisons. Integration of biomarker-driven patient selection—including molecular profiling, ctDNA/utDNA, and target expression such as FGFR, Nectin-4, and fibroblast activation protein—should be prioritized to better define treatment responsiveness and optimize trial design. In addition, incorporation of clinically relevant endpoints such as renal function preservation, quality of life, and durable response rates will be essential, particularly in the context of kidney-sparing approaches.

## 4. Conclusions

Upper-tract urothelial carcinoma remains a rare but aggressive malignancy in which therapeutic decisions must balance oncologic control with preservation of renal function. Radical nephroureterectomy continues to anchor management of high-risk localized disease, yet contemporary evidence now supports a more integrated approach. Adjuvant platinum-based chemotherapy has demonstrated a clear disease-free survival benefit in high-risk patients, and prospective studies of neoadjuvant cisplatin-based regimens suggest that earlier delivery of systemic therapy, before postoperative renal decline, may improve long-term outcomes. In advanced urothelial carcinoma, treatment paradigms have been transformed by chemoimmunotherapy, maintenance checkpoint inhibition, FGFR targeted therapy, and antibody–drug conjugates, all of which included patients with UTUC and are now embedded in routine practice. Looking ahead, biomarker-driven risk stratification with circulating tumor DNA, expansion of kidney-sparing strategies through chemoablation and photodynamic therapy, novel intraluminal drug-delivery platforms, and emerging theranostic approaches such as radioligand therapy represent important frontiers. The central challenge remains the generation of UTUC specific prospective data to refine patient selection and optimize sequencing, ensuring that innovation translates into durable survival gains without compromising renal function.

## Figures and Tables

**Table 2:** Adjuvant therapy studies including patients with upper-tract urothelial carcinoma.

Trial	Total N	UTUC n (%)	Population	Intervention	Comparator	Primary Endpoint	Key Result
**POUT** [23]	261	261 (100%)	pT2–T4 or pN+ after radical nephroureterectomy	Cisplatin or carboplatin + gemcitabine	Surveillance	Disease-free survival	HR 0.55 for DFS; HR 0.68 for OS (95% CI, 0.46 to 1.00)
**CheckMate 274** [24]	709	149 (21%)	High-risk muscle-invasive urothelial carcinoma after radical surgery (ypT2-ypT4 or ypN1 OR pT3-4 or pN1)	Nivolumab	Placebo	Disease-free survival	HR for DFS 0.71(95% CI, 0.58 to 0.86; HR for OS 0.76 (95% CI, 0.61 to 0.96)
**IMvigor 010** [27]	809	54 (7%)	High-risk muscle-invasive urothelial carcinoma after radical surgery (ypT2-4 or ypN+ OR pT3-4 or pN+)	Atezolizumab	Observation	Disease-free survival	HR for DFS 0·89 (95% CI, 0·74 to 1·08)
**AMBASSADOR** [26]	702	178 (25%)	High-risk muscle-invasive urothelial carcinoma after radical surgery (≥ypT2 or ypN+ or R1 OR pT3 or pN+ or R1)	Pembrolizumab	Observation	Disease-free survival	HR for DFS 0.73; 95% CI, 0.59 to 0.90

## Data Availability

Data are contained within the article
